# The Effects of Urethane on Rat Outer Hair Cells

**DOI:** 10.1155/2016/3512098

**Published:** 2016-12-05

**Authors:** Mingyu Fu, Mengzi Chen, Xiao Yan, Xueying Yang, Jinfang Xiao, Jie Tang

**Affiliations:** ^1^Department of Physiology, School of Basic Medical Sciences, Southern Medical University, Guangzhou 510515, China; ^2^Department of Anesthesiology, Nanfang Hospital, Southern Medical University, Guangzhou 510515, China; ^3^Department of Anesthesiology, Sun Yat-sen Memorial Hospital, Guangzhou 510120, China

## Abstract

The cochlea converts sound vibration into electrical impulses and amplifies the low-level sound signal. Urethane, a widely used anesthetic in animal research, has been shown to reduce the neural responses to auditory stimuli. However, the effects of urethane on cochlea, especially on the function of outer hair cells, remain largely unknown. In the present study, we compared the cochlear microphonic responses between awake and urethane-anesthetized rats. The results revealed that the amplitude of the cochlear microphonic was decreased by urethane, resulting in an increase in the threshold at all of the sound frequencies examined. To deduce the possible mechanism underlying the urethane-induced decrease in cochlear sensitivity, we examined the electrical response properties of isolated outer hair cells using whole-cell patch-clamp recording. We found that urethane hyperpolarizes the outer hair cell membrane potential in a dose-dependent manner and elicits larger outward current. This urethane-induced outward current was blocked by strychnine, an antagonist of the *α*9 subunit of the nicotinic acetylcholine receptor. Meanwhile, the function of the outer hair cell motor protein, prestin, was not affected. These results suggest that urethane anesthesia is expected to decrease the responses of outer hair cells, whereas the frequency selectivity of cochlea remains unchanged.

## 1. Introduction

Under general anesthetics, decreased hearing sensitivity is common in both animal research and clinical settings. Several studies have demonstrated that different anesthetics increase auditory brainstem response thresholds [1–3] and depress neural excitability in the auditory midbrain [4, 5] and cortex [6–8]. The sensitivity of the auditory system could be changed by anesthetics at two levels: the cochlea and the auditory neurons. Because any change in cochlear function may influence the response of central auditory neurons, the effects on the cochlea are essential for the anesthetic-induced reduction in hearing sensitivity. However, the majority of studies have focused on the neural responses, whereas few have examined cochlear function [1]. Urethane has been widely used in animal research for more than a century because it exerts minimal effects on the cardiovascular and respiratory systems. Although urethane has been reported to depress the sound-evoked activity of the auditory system [4–6], its direct effect on the cochlea, particularly sensory hair cells, remains unknown.

Sensory hair cells in the cochlea not only translate sound vibration into electrical impulses but also amplify the signals of low-level sound. The latter process, defined as cochlear amplification, confers incredible sensitivity on mammalian hearing in a tremendous intensity range [9]. Cochlear amplification in mammals is attributed to outer hair cells (OHCs), which can alter their somatic length on the order of micrometers in response to membrane potential changes [10]. This electromotility is powered by the unique motor protein, prestin, on the OHC lateral membrane. The voltage-dependent structural conformation of prestin drives OHC somatic motility, which regulates cochlear amplification [11, 12]. However, the voltage-to-length change conversion function (Δ*L*-*V*) of OHCs is nonlinear and asymmetric: depolarization produces larger cell length changes than comparable hyperpolarization [13–15]. Therefore, the changes in the membrane potential may alter the operating point on the Δ*L*-*V* function and influence the overall level of cochlear amplification.

Because their electromechanical conversion occurs via a feedback mechanism, OHCs play a critical role in the efferent gain control of the cochlear amplifier. OHCs are innervated by efferent fibers that originate in the superior olivary complex [16, 17]. These efferent fibers form synapses at the base of the OHCs and use acetylcholine (ACh) as their primary neurotransmitter [18, 19]. Nicotinic ACh receptors (AChR) have been identified on OHCs [18, 20, 21], and ACh hyperpolarizes the membrane potential of isolated OHCs [22]. The efferent activity of the olivocochlear nerve bundle during electrical stimulation has been shown to be inhibitory [23], thereby reducing the gain of the cochlear amplifier and providing protection to the ear against overstimulation [24]. A pharmacological study has indicated that urethane enhances the function of nicotinic AChRs while inhibiting the responses of NMDA and AMPA receptors [25]. We hypothesize that urethane influences the micromechanics of the organ of Corti via the OHCs and, in turn, the cochlear amplification process. If so, urethane anesthesia provides an alternative strategy to modulate cochlear amplification. By comparing the cochlear microphonic (CM) responses between awake and urethane-anesthetized rats, we found that the activity of OHCs was significantly reduced by urethane. We also measured the membrane potential and current as well as prestin activity in isolated OHCs in the presence of urethane. Our results indicate that urethane hyperpolarizes the OHC membrane potential, which is at least partially mediated by the AChR. However, prestin activity remains intact.

## 2. Materials and Methods

All experimental preparations, surgeries, and protocols used in this study were approved by the Animal Care and Use Committee of Southern Medical University of China. Healthy young Sprague Dawley rats of either sex (21–28 days old, body weight 40–70 g) exhibiting normal hearing were used for the experiments. The CM measurements and whole-cell patch recordings were performed as previously described [26, 27]. These methods are briefly described as follows.

### 2.1. CM Measurements in Awake Rats

**Figure 1:**
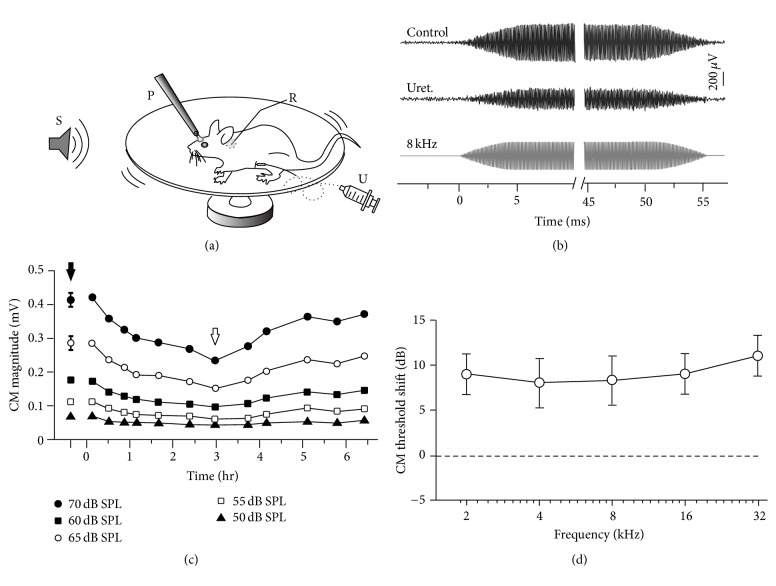
Reduction in the CM after urethane anesthesia. (a) A schematic of our CM recording setup. S, sound stimulation delivered by a speaker; P, metal post for head fixation; R, silver wire recording electrode; U, urethane delivery. The rat was awake and allowed to run freely on a rotatable plate. (b) Examples of the CM measured in response to an 8 kHz, 70 dB SPL tone burst (sound waveform shown in the bottom panel). The magnitude before urethane injection (upper panel, filled arrow in (c)) compared to that after anesthesia (middle panel, open arrow in (c)). (c) The time course of the CM magnitude changes after urethane injection. The curves shown are CM responses to different sound levels. Three CM measurements evoked before urethane injection were averaged as a control (mean ± SD) for each sound level. Time 0 indicates the time point of urethane injection. (d) CM threshold shifts for urethane application. Data presented as mean ± SD. Note the CM thresholds were elevated at all frequencies examined (*p* < 0.05, Student's *t*-test, *n* = 5).

Three days before recording, the rats were anesthetized using sodium pentobarbital. The scalp was removed, and a metal screw was mounted on the skull using glass ionomer cement. The animals were subcutaneously injected with 0.1 mg/kg buprenorphine and returned to their home cages to recover. During the recovery period, the animals were trained to become accustomed to being head-fixed in the recording setup. To fix the head, the screw was tightly clamped to a metal post. The rat was able to run freely on a plastic plate rotating around its center as described in our recent study [28]. On the day of recording, surgery was performed in a sound-proof chamber. The rats were anesthetized with 1.5% isoflurane. Then, the head was fixed to the metal post. A small incision was made via a dorsolateral approach to the pinna to expose the acoustic bulla. A silver wire recording electrode (tip diameter, ~500 *μ*m) was placed near the round window membrane through an opening of 3 mm in diameter on the acoustic bulla (Figure 1(a)). The animal was allowed to recover from isoflurane anesthesia for at least 30 min. To acquire the CM under awake conditions, the recording was initiated after the animal exhibited normal running. Then, the animal was intraperitoneally injected with 1 g/kg urethane using a pipette to examine the effects of this anesthetic. The entire recording session lasted for approximately 5–10 hours.

Tone bursts (50 ms duration, 5 ms rise/fall time) of various frequencies (2, 4, 8, 16, or 32 kHz) and intensities (0–70 dB SPL at 5 dB intervals) were presented using a calibrated TDT ES1 speaker located 50 cm away from the recorded ear. The frequency-amplitude scan was computer controlled (TDT System 3, Tucker-Davis Technologies) and was delivered in a randomized sequence. Each frequency-amplitude combination was repeated 10 times. The CM responses to the tone bursts were amplified, filtered, and recorded using an A/D converter (1440A/700B system, Molecular Devices). The noise level of the recording system was approximately 10 *μ*V. Customized MATLAB software was used for offline data processing, such as response averaging and response amplitude extraction.

### 2.2. Cell Isolation

The animals were anesthetized (CO_2_ inhalation) and decapitated, and the inner ears were rapidly removed from the temporal bones and placed in Leibovitz's L-15 media (Invitrogen, Carlsbad, CA). The organ of Corti was isolated from the middle and apical turns of the cochlea. After mild enzymatic digestion for 5 min (2 mg/mL collagenase IV, Sigma, St. Louis, MO) and gentle pipetting, the cells were transferred to a small plastic chamber filled with enzyme-free culture medium (~1.5 mL). The standard medium was Leibovitz's L-15, supplemented with 10 mM HEPES (Invitrogen, Carlsbad, CA) and adjusted to pH 7.35 and 300 mOsm. Then, the chamber containing the cells was placed on the stage of an inverted microscope (Nikon, Eclipse FN1) equipped with a video camera. Healthy-appearing solitary OHCs were selected for the electrophysiological experiments if they displayed no obvious signs of shrinkage, swelling, damage, or deterioration such as granularity or translocation of the nucleus.

### 2.3. Whole-Cell Patch-Clamp Recordings

These experiments were performed at room temperature (22 ± 4°C) under video monitoring. The OHCs were bathed in L-15 medium buffered with 10 mM HEPES (pH 7.35, 300 mOsm). An Ag/AgCl ground electrode was installed in the bath. The patch electrodes were pulled from 1.5 mm glass capillary tubes at resistances between 3 and 6 M*Ω* using a horizontal micropipette puller (Model P-97, Sutter). The electrodes were back-filled with a solution containing (in mM) 145 KCl, 2 MgCl_2_, and 10 HEPES. The access resistance typically ranged from 10 to 17 M*Ω* when the whole-cell recording configuration was established. At least 80% of the access resistance was compensated. In most of our recordings, the whole-cell currents were less than 3 nA.

Under computer control, hyperpolarizing and depolarizing voltage steps (250 ms duration and ranging from −140 to +94 mV in 13 mV increments) were used to elicit whole-cell currents. The low-pass-filtered currents (corner frequency of 5 kHz) were amplified using an Axopatch 200B amplifier (Axon Instruments). The urethane-evoked current responses were recorded in voltage-clamp mode. To obtain large urethane-evoked outward currents, the cells were typically held at 0 mV. The whole-cell currents and evoked current responses were acquired using pClamp 10 software (Molecular Devices) on a computer connected to an A/D converter (Digidata 1322A, Axon Instruments). The sampling frequency was between 5 and 10 kHz. The data were analyzed using the pClamp software package.

For nonlinear capacitance (NLC) measurements, the whole-cell patch-clamp technique was performed as described above. The membrane capacitance was measured using a two-sine-wave voltage stimulus protocol (10 mV peak at both 390.6 Hz and 781.2 Hz) with subsequent fast Fourier transform-based admittance analysis [29] at a holding potential of 0 mV. The data were acquired using jClamp software (Scisoft, New Haven, CT) and were analyzed using OriginPro software (OriginLab Corporation, Northampton, MA).

The NLC can be described as the first derivative of a two-state Boltzmann function of nonlinear charge movement to voltage [30]. The capacitance function is described as

(1) Cm=Clin+Qmaxαexp⁡αVm−V1/21+exp⁡−αVm−V1/22.

Four parameters (*Q*_max_, *V*_1/2_, *C*_lin_, and *z*) from the equation were obtained: *Q*_max_ is the maximum charge transfer; *V*_1/2_ is the peak of the voltage-dependent capacitance; *C*_lin_ is the linear capacitance; and *α* = *ze*/*kT* is the slope of the voltage dependence of the charge transfer. Furthermore, *k* is the Boltzmann constant, *T* is absolute temperature, *z* is the valence of the charge movement, and *e* is the electron charge. *C*_lin_ is the linear capacitance representing the surface area of the membrane (i.e., the cell size). To compare the magnitude of the NLC and *Q*_max_ obtained from different cells of varying size, we normalized the NLC and *Q*_max_ to *C*_lin_.

### 2.4. Drug Application

The drugs were dissolved in standard medium (L-15) adjusted to pH 7.35 and 300 mOsm. All solutions were freshly prepared from stock solution before each experiment. Urethane was delivered via pressure ejection from a micropipette with a tip diameter of ~5 *μ*m positioned 20–50 *μ*m from the bottom of the cell. The duration and strength of the pressure were controlled using a homemade microinjector. Care was taken to assure that the application of a drug solution did not alter the position of the cell or influence the measurements. In the strychnine coapplication experiments, the strychnine solution was slowly perfused into the bath (1 mL/min) without disturbing the position of the cells. The entire bath was exchanged when strychnine was applied. Urethane was dissolved in strychnine solution and delivered via pressure ejection as described above. All of the drugs were applied to achieve a final concentration until a consistent response was observed and a washout was performed after each application.

### 2.5. Data Analysis

Results are presented as the mean ± SD. A Student's *t*-test was used to examine the significance of the difference between the responses obtained before and during the drug applications. Significance was determined as *p* < 0.05. Excel software and OriginPro software were used for calculating, data fitting, and plotting.

## 3. Results

### 3.1. The CM under Urethane Anesthesia

The inner and outer hair cells, which are the sensory receptor cells of the inner ear, function as a transducer by converting the mechanical movement of the basilar membrane into an alternating electrical voltage. This alternating voltage is defined as the CM, which mimics the waveform of a sound stimulus. Representative CM recordings are shown in Figure 1, in which the effects of urethane anesthesia are presented for the same rat. We performed CM recordings on head-fixed awake rats to monitor the receptor potential before and after urethane application (Figure 1(a)). To avoid the middle ear reflex, mild tone bursts (less than 70 dB SPL) were used to elicit the CM responses.

As shown in Figure 1(b), an 8 kHz tone (level at 70 dB SPL) evoked a CM at an amplitude of ~424 *μ*V. The amplitude of the CM responses at saturation levels was reduced by 44% to 238 *μ*V after the intraperitoneal injection of urethane. The proximity of the recording electrodes to the hair cells may affect the recorded amplitudes. To ensure that our control recordings were not influenced by the activity of the animal, we averaged at least three evoked CM measurements before the urethane injection. For the CMs measured from all five rats, urethane induced a significant decrease in the CM of 39.3% on average (*p* < 0.01, Student's *t*-test).

The time course of this urethane effect was examined in five rats that exhibited at least an 80% recovery in the CM amplitude. The representative changes in the CM over time after urethane injection are shown in Figure 1(c). The initial decrease in the CM was observed ~25 min after the urethane injection, reached its lowest value within ~3 hours, and then recovered gradually. The time of the peak reduction was highly variable between different rats, ranging from 45 min to 3 hours (45 min, 75 min, 90 min, 3 hours, and 3.2 hours, resp.). The CM responses to different sound levels were also measured. As shown in Figure 1(c), the changes in the CM amplitudes at different sound levels followed a similar time course. We compared the CM thresholds before and after urethane application (Figure 1(d)). The CM thresholds were defined as the minimum sound level that evoked a detectable CM response. Consistent with the CM magnitude measurements, the CM thresholds were increased by urethane application at all frequencies examined. The increase of threshold is similar and shows no significance at different sound frequencies (*p* > 0.05, Student's *t*-test).

### 3.2. OHC Responses under Current and Voltage Clamp

The CM response is dominated by the OHCs in the organ of Corti [9, 31]. As such, the CM reduction in our experiments represents a significant reduction in OHC activity after urethane application. To determine how urethane alters the responses of OHCs, whole-cell current- and voltage-clamp recordings were performed from OHCs acutely isolated from the middle and apical turns of the rat cochlea. Isolated OHCs can easily be identified based on visual inspection: the OHCs display a cylindrical morphology with a nucleus located near the base, whereas inner hair cells are flask-shaped with an upper nucleus position. Another indication of OHCs is a functional characteristic: the visible motile responses elicited by the rapid membrane potential changes generated during our patch-clamp measurements [22, 32].

**Figure 2:**
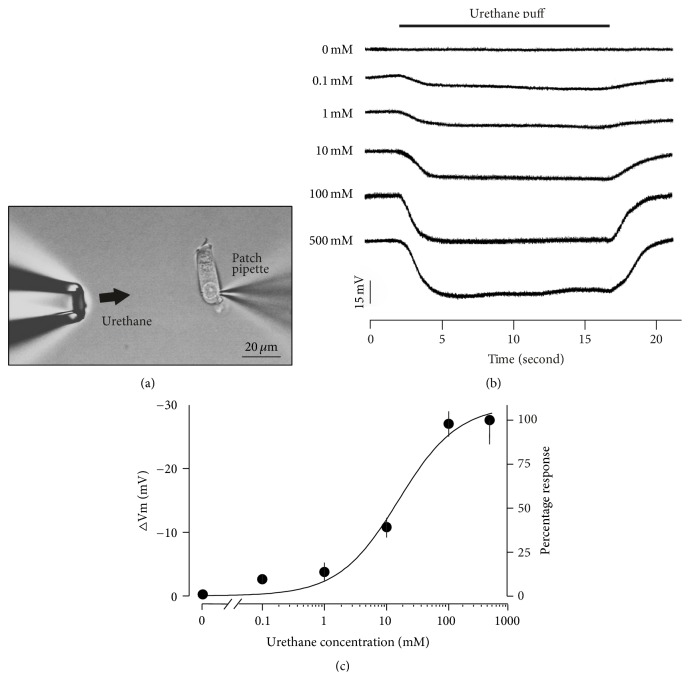
The urethane-induced hyperpolarization of the OHC membrane potential. (a) A photograph showing the experimental setup for the whole-cell patch-clamp recordings. Urethane was delivered via pressure ejection from a micropipette positioned ~50 *μ*m from the cell. The microphotograph was captured using an upright microscope under bright field illumination. The bar represents 20 *μ*m. (b) Examples of membrane potentials recorded from OHCs clamped to zero membrane current during the delivery of a urethane puff (0.1 mM, 1 mM, 10 mM, 100 mM, or 500 mM concentration, timing denoted by the horizontal bar) or standard medium (0 mM). (c) The dose-response curve of the OHC membrane potential evoked by 0 mM (obtained from 4 cells), 0.1 mM (5 cells), 1 mM (3 cells), 10 mM (5 cells), 100 mM (5 cells), or 500 mM (4 cells) urethane. The data are presented as the mean values; the error bars represent the SD. The values are also normalized to the mean reduction evoked by 500 mM urethane. The smooth curve is the Hill equation with a half-activating concentration of 15.4 mM and a slope of 0.91.

The average zero current membrane potential under whole-cell recording conditions was −54 mV (SD = 7 mV, *n* = 19). To determine the influence of urethane, different concentrations of urethane were applied to the recorded OHC via local perfusion for ~15 s (as shown in Figure 2(a)). As a control, recordings were also performed with L-15 medium: no membrane potential change was detected during L-15 perfusion (0 mM in Figure 2(b)). The stability of this recording indicates that our measurements were not affected by the perfusion flow rate. When 100 mM urethane was delivered to an OHC clamped at 0 nA, the steady-state membrane potential was hyperpolarized by 28.6 mV and was repolarized shortly after drug application (Figure 2(b)). This reversible hyperpolarization was detected in all five OHCs measured (mean ± SD = 27.0 ± 3.9). To rule out the effects of urethane on the OHC membrane potential, we applied several concentrations of urethane. Urethane at a concentration as low as 0.1 mM, which is ~1/100 of the dose typically used to anesthetize animals, elicited a detectable membrane hyperpolarization.  Figure 2(c) displays the average membrane potential changes induced by different urethane concentrations. These data also provide the mean normalized response. The smooth curve represents a fit according to the following form of the Hill equation: *V*_Uret_ = 100/[(*K*_*D*_/[Uret])^*n*^ + 1]. A fifty percent reduction in the membrane potential was detected at 15.4 mM (*K*_*D*_), and the slope (*n*) of the membrane potential change to the urethane concentration was 0.91. Subsequent experiments used 100 mM urethane because this concentration consistently evoked an apparent response.

**Figure 3:**
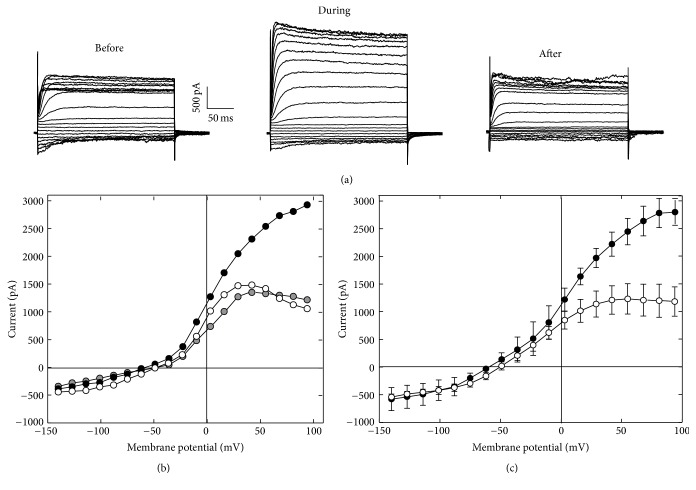
Urethane-induced membrane current changes. (a) Example of the membrane current waveforms recorded from a solitary OHC before, during, and after 100 mM urethane application. The cell was held at −70 mV, and voltage commands varied from −140 to +94 mV in 13 mV steps. (b) The *I*-*V* curves derived from the steady-state responses shown in (a). The open, black, and gray filled circles represent the responses before, during, and after urethane application, respectively. (c) Plot of the average (±SD) *I*-*V* curves recorded from OHCs (*n* = 11) before (open circles) and during (filled circles) urethane application.

We also examined the effects of urethane on the membrane current by using voltage-clamp recordings. A representative example of the whole-cell current recorded from an isolated OHC is shown in Figure 3(a). When the membrane potential was clamped from −140 to +94 mV, the cell currents changed from −445 pA (inward) to +1480 pA (outward), resulting in a dynamic range of 1925 pA. The response measured under control conditions is consistent with results previously published for guinea pig [22, 33] and gerbil OHCs [34]. The local perfusion of 100 mM urethane significantly increased the current magnitudes, especially at potentials greater than −50 mV.  Figure 3(b) shows the current-voltage (*I*-*V*) curve derived from the steady-state responses shown in Figure 3(a). The dynamic range was approximately 72% larger after urethane application in the example presented. The mean change of *I*-*V* curves recorded from 11 OHCs was shown in Figure 3(c). Notably, the increased outward currents occurred at high membrane potentials.

### 3.3. The Effect of Strychnine on the Urethane-Induced Response

Urethane is not an endogenous neurotransmitter or modulator. Therefore, it is unlikely that the effects of urethane are mediated by a specific urethane receptor. The apparent changes in the OHC current response in the voltage-clamp experiments are most likely due to the effects of urethane on existent ion channels. Acetylcholine is the primary efferent neurotransmitter in the cochlea and is released from the efferent chemical synapses at the base of the OHCs [19, 24]. The *α*9 subunit of the nicotinic AChR family, which was identified from a rat genomic library, has been demonstrated to play an important role in the ACh-induced responses of OHCs [18, 20, 21]. To determine whether the urethane-induced response occurs via this AChR, we examined the effect of strychnine (a potent antagonist of the *α*9 AChR subunit) on the urethane-induced responses. Because the membrane current is directly related to nicotinic AChR activity and is easy to record, we measured the urethane-induced current as an indicator of nicotinic AChR activity.

**Figure 4:**
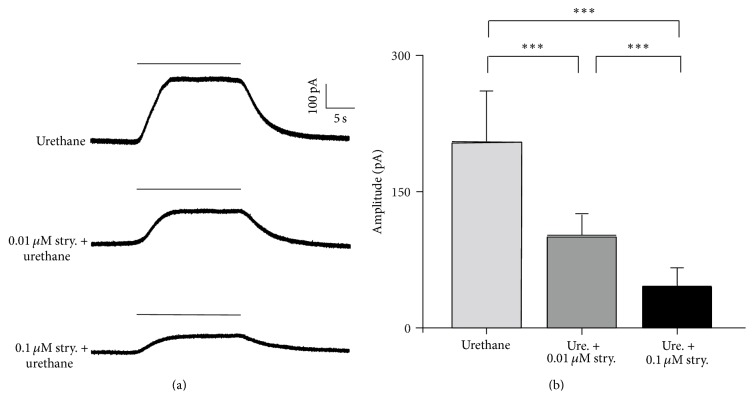
Urethane induces membrane current changes via the nicotinic AChR. (a) Strychnine, an antagonist of *α*9 AChRs, partially blocked the OHC response to urethane. The cell was held at 0 mV, and 100 mM urethane was pressure-ejected near the cell (timing denoted by the horizontal bar) to obtain the control response (top trace). Either 0.01 (middle trace) or 0.1 *μ*M strychnine (bottom trace) was coapplied with urethane to the OHC. The magnitude of the outward currents decreased as the coapplied strychnine concentration increased. A 3 min waiting period was used between each trial. (b) The average amplitudes of outward current in response to 100 mM urethane or 100 mM urethane coapplied with two different doses of strychnine. Bar = SD. ^*∗∗∗*^ *p* < 0.001, Student's *t*-test. *N* = 7.

To obtain the urethane-induced current, the membrane potential of isolated OHCs was held at 0 mV. The top trace in Figure 4(a) shows a 280 pA upward change of membrane current that correlated in time to the perfusion of 100 mM urethane onto this cell (~20 s). As indicated in Figure 3(b), urethane increased the outward current at a membrane potential of 0 mV. Therefore, the magnitude of the observed current change reflects the amplitude of the outward current elicited by urethane. Coapplication of 0.01 *μ*M strychnine reduced the amplitude of the urethane-induced current to 114 pA at saturation level (Figure 4(a), middle trace). These data suggest that the urethane-induced response is mediated, at least in part, via AChR assembled from *α*9 subunits. Then, a higher concentration of strychnine (0.1 *μ*M) was coapplied to examine whether the urethane response was blocked in a dose-dependent manner. To minimize desensitization of the receptor, 3 min washout was set between two concentrations. As shown in the bottom trace (Figure 4(a)), the urethane-induced outward current was further reduced to 48 pA in the presence of 0.1 *μ*M strychnine. In the seven OHCs measured (Figure 4(b)), the magnitude of the urethane-induced response was significantly reduced by the coapplication of both 0.01 *μ*M (by 54 ± 12% on average) and 0.1 *μ*M strychnine (by 79 ± 10% on average) (both *p* < 0.001, Student's *t*-test).

### 3.4. NLC Measurement during Urethane Treatment

Mammalian OHCs contract or elongate at acoustic frequencies depending on the membrane potential of the cell [10, 35, 36]. This process, defined as electromotility, is necessary for cochlear amplification [37–39]. Prestin, a unique voltage-dependent motor protein found in the membrane of OHCs, mediates the electromotility of OHCs [11, 40]. The voltage-sensing and motor functions of mammalian prestin manifest as two characteristics: the NLC and electromotility. The NLC and electromotility are fully coupled in mammals [30, 41, 42] and can be characterized using a simple two-state Boltzmann function. Because the NLC can be easily and accurately measured experimentally, we measured the NLC to evaluate the effects of urethane on prestin function.

**Figure 5:**
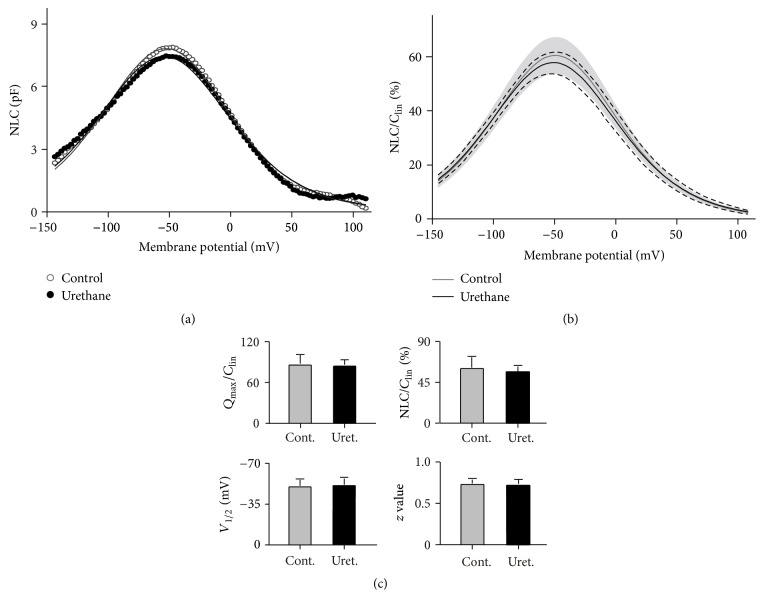
The effects of urethane on the NLC measured from OHCs. (a) The NLC obtained from a representative OHC before (control, open circles) and during (urethane, filled circles) 100 mM urethane application. The capacitance-voltage responses were fitted to the Boltzmann function (shown as the gray and black lines). *C*_lin_ was subtracted from the NLC. pF, picofarads. (b) Pooled data of the NLCs recorded from 10 OHCs. The NLCs were normalized to the corresponding *C*_lin_, and the curves were plotted as the mean NLC ± SD. The SD around the mean is indicated by the shaded region for the control and by dashed lines for urethane treatment. (c) Four parameters derived from the curve fit to the Boltzmann function. The data are expressed as the means and the SDs; *N* = 10. No significant difference was detected between the cases before and during urethane application (Student's *t*-test, *p* > 0.05).

Figure 5(a) shows an example of the NLC obtained from an isolated OHC under the whole-cell patch-clamp configuration. As shown in the control (open circles and gray line) treatment before urethane application, the NLC is characterized by a bell-shaped dependence on the membrane potential and a peak at −54.8 mV for this cell. No clear change was detected in response to application of 100 mM urethane (filled circles and black line). We examined the NLC from a total of 10 OHCs in response to urethane treatment.  Figure 5(b) presents the mean and SD of the normalized NLC from these cells. Four parameters (*Q*_max_, *C*_lin_, *V*_1/2_, and *z*) were obtained from a curve fit of the NLC response using the first derivative of the Boltzmann function (heavy lines in Figures 5(a) and 5(b)). The normalized mean values and SDs of the four parameters from the 10 OHCs are plotted in Figure 5(c). No statistically significant difference was found in response to urethane treatment for all parameters (*p* > 0.05, Student's *t*-test).

## 4. Discussion

We have shown for the first time that urethane affects the electrical response properties of isolated OHCs. As shown in Figure 2, urethane hyperpolarizes OHCs by approximately 30 mV. Our voltage-clamp data shows that when OHCs were depolarized, the outward current was significantly increased by urethane (Figure 3). This effect was voltage-dependent: it was more pronounced at membrane potentials higher than −50 mV. In mature OHCs, two currents are primarily involved in this process: (1) the voltage activated outward K^+^ current and (2) the Ca^2+^-activated K^+^ current. The voltage-dependent K^+^ current is activated at membrane potentials from −90 mV to −50 mV, displaying half activation at −80 mV [43]. Because the urethane-induced current change was clearly detected at membrane potentials >−50 mV, its effect is likely not via this channel.

The Ca^2+^-activated K^+^ channel was first reported by Ashmore and Meech [44]. At membrane potentials >−35 mV, this K^+^ channel is opened by an influx of Ca^2+^, leading to K^+^ efflux [45]. Under physiological conditions, the activation of AChRs causes an influx of Ca^2+^ [22]. We assumed that the effect of urethane on OHCs involves this process based on our strychnine experiment. The *α*9 subunit of the nicotinic AChR family has been demonstrated to be the primary nicotinic AChR subunit in OHCs [20]. The *α*9 subunit displays unique pharmacological properties similar to those detected in cochlear hair cells. We found that strychnine, a potent antagonist of *α*9-containing AChRs, significantly blocks the urethane-induced outward current (Figure 4). Therefore, we propose that urethane activates *α*9-containing AChRs and induces a Ca^2+^ influx. Then, this influx of Ca^2+^ leads to the opening of Ca^2+^-activated K^+^ channels and subsequent K^+^ efflux, resulting in hyperpolarization of the OHCs. Consistent with our results, a study in* Xenopus* oocytes indicates that urethane enhances the response of nACh receptor [20]. Our data is also supported by the urethane effects on cochlear function by measuring DPOAE [46]. Urethane decreases the efferent influence from medial olivocochlear terminus to OHCs via the *α*9 receptor. The presence of urethane may reduce the effects of ACh release from efferent fibers. However, the direct mechanism underlying nicotinic AChR activation by urethane remains unknown.

The cylindrically shaped OHCs alter their cell length in response to membrane potential changes, exhibited as either a somatic elongation (upon hyperpolarization) or contraction (upon depolarization). This somatic motility of OHCs is responsible for cochlear amplification, which contributes to the exquisite frequency selectivity and sensitivity in mammals [10, 37]. However, this change in length is asymmetric: the magnitude of contraction is much larger than that of elongation [13–15]. Figure 2 shows that urethane hyperpolarizes OHCs by approximately 30 mV. This potential change moves the operating point of the voltage-to-length change conversion function toward lower slope, thereby reducing the overall augmentation of OHC electromotility. Based on the voltage-to-length change conversion function for OHCs in the guinea pig, a 30 mV hyperpolarization reduces the total motility magnitude by approximately 25–35% [15]. We expect a corresponding maximal magnitude change in rat OHCs.

However,* in vitro* acetylcholine application evokes an increase of electromotile responses of isolated OHCs that develops on a time scale of several seconds [22]. It would increase the driving force for the mechanotransduction current, causing the increase of cochlear microphonic potential. It seems a mismatch between our* in vitro* data and* in vivo* CM results. For* in vitro* experiment, a high concentration urethane was applied to isolated OHCs directly. Therefore, the effects were observed in seconds. However,* in vivo*, a safe concentration urethane was injected intraperitoneally. It may take time for urethane to reach and accumulate in the inner ear. The time course for OHCs may differ from that for cardiovascular and respiratory systems. In addition to a change in the membrane potential, mechanical properties of OHCs could also influence the cochlear amplifier. It is the consensus that normal cell morphology and somatic stiffness of OHCs are essential for cochlear amplification [12, 47]. Acetylcholine decreases the axial stiffness of the OHCs and reduces the overall mechanical load of the aggregate OHC, resulting in the increase of motile magnitude [22]. It has been suggested that the changes of the OHCs stiffness are mediated by Ca^2+^-dependent phosphorylation of the OHC cortical cytoskeleton [22, 48]. Since urethane would also activate *α*9-containing AChRs and induce a Ca^2+^ influx, a similar decrease of OHCs axial stiffness and a reduction of global cochlear activity may be induced by urethane application* in vivo*.

The electromotility of OHCs is presumably attributed to the voltage-dependent activity of the motor protein prestin [11, 40]. A generally accepted model for prestin function is that intracellular anions (in most cases Cl^−^) move toward the extracellular surface upon hyperpolarization and toward the cytoplasmic side in response to depolarization [40]. This anion translocation produces a nonlinear change in the membrane capacitance and, subsequently, triggers a conformational change in prestin, which ultimately alters the somatic length of the OHC. According to this model, any changes in this voltage-dependent activity of prestin may alter the motility of OHCs and affect the overall level of cochlear amplification. For all measured parameters reflecting the properties of prestin function, we did not observe any significant change in response to urethane in our experiments (Figure 5). Therefore, we assumed that the influence of urethane was not the alteration of the motile activity of prestin. Nonetheless, anion transfer may be significantly reduced by urethane-induced hyperpolarization. In mammalian OHCs, this charge movement is represented as the NLC, which is characterized by a bell-shaped dependence on the membrane potential that peaks between −70 and −20 mV (−50 mV in our results) [30, 41]. The urethane-induced hyperpolarization shifts the operating range of the membrane potential away from this peak, thus reducing the charge movement and the activity of prestin. This effect is a likely mechanism by which urethane reduces OHC motility* in vivo*. Because the electromotility of OHCs feeds a cycle-by-cycle force to the organ of Corti so that the sound vibration is amplified, it is conceivable that even a modest reduction in the motility of OHCs could reduce the overall level of cochlear amplification.

The results from isolated OHCs are consistent with our* in vivo* experimental evidence (Figure 1). Although the time courses of the urethane-induced effects were different, all of the rats examined exhibited a similar reduction in the CM magnitude. Despite the expanding research to awake animal models of monkeys [49, 50], bats [51, 52], mice [28], and rats [53], the majority of auditory studies are based on the results obtained from anesthetized animals. The frequency selectivity of auditory neurons is composed of two elements: the frequency tuning of the cochlea and the refinement of the auditory neural system. The detected anesthesia-induced changes in neural responses involve the effects of the anesthetic on not only the neurons themselves but also the peripheral receptors (hair cells). Therefore, it is critical to identify the influence over hair cells from the integrity. Our data show that urethane elicited a ~10 dB CM threshold lifting (Figure 1(d)), which indicates that the depression of OHCs leads to a reduction in hearing sensitivity. Furthermore, this depression is identical at all sound frequencies, suggesting that urethane affects OHCs along the entire basilar membrane. These results are similar to those of isoflurane and ketamine, which have been assessed using distortion product otoacoustic emissions [1].

## 5. Conclusions

The present study found that urethane hyperpolarizes outer hair cells, resulting in a reduction in hearing sensitivity without affecting frequency selectivity. Our findings indicate that anesthetics directly affect cochlear hair cells and provide an alternative strategy to modulate cochlear functions.
